# Can Non-Pharmacological Treatment Promote Additional Benefit for Children with Familial Hypercholesterolemia Treated with Statins?

**DOI:** 10.5935/abc.20180235

**Published:** 2018-12

**Authors:** Luiza Antoniazzi

**Affiliations:** Instituto do Coração (InCor), São Paulo, SP - Brazil

**Keywords:** Child, Dietary Fats, Diet, Hyperlipoproteinemia Type II, Hypercholesterolemia/blood, Statins/therapy, Hydroxymethylglutaryl-CoA Reductase Inhibitors

Familial hypercholesterolemia (FH) is described as an autosomal dominant hereditary disease characterized by elevation of total cholesterol and low density lipoprotein (LDL-c).^1^

FH is considered a major modifiable risk factor for the development of atherosclerosis and cardiovascular disease (CVD).^2^ The early institution of lipid-lowering therapy and its lifelong maintenance are important aspects in the prevention of premature CVD and the risk of death in this population, increasing life expectancy in these patients.^3^

The current guidelines^4-7^ recommend, pharmacological treatment for individuals aged 8 to 10 years. It should only be used for younger children with extreme elevation of LDL-c and associated risk factors. Radaelli et al.^8^ performed a meta-analysis with ten randomized clinical trials conducted with children and adolescents from 8 to 18 years of age who underwent therapy with statins for FH. They showed the statins significantly reduced LDL-c in children with FH.

This study contributed to the evaluation of the effectiveness of lipid-lowering therapy in children with FH. However, there are no data on efficacy and safety in the long term. The included studies ranged from 12 to 104 weeks and considering that individuals with FH will need lifelong treatment, it is extremely important that safety studies of different types of treatment be carried out with longer study times.

The importance of drug treatment to avoid unfavorable outcomes in individuals with FH should be considered, but care should be broader and include good detection strategies as well as the implementation of non-pharmacological treatment.

The most cost-effective strategy for FH diagnosis is the screening of mutations in first degree relatives of individuals identified with FH.^9^ In screening rounds, first degree relatives identified with FH become the index cases and their relatives are traced. This is referred to as cascading genetic screening. The molecular diagnosis of FH can, in addition to identifying affected relatives, allows them to receive the adequate treatment. Children are the biggest beneficiaries of the screening program as they have the possibility of initiating treatment before high cholesterol levels have caused a high degree of atherosclerosis.^3^

The consensus of the European Atherosclerosis Society^4^ and the I^st^ Brazilian Guideline for Familial Hypercholesterolemia^1^ is that dietary treatment is required in addition to pharmacological treatment of patients with FH.^10,11^

The nutritional treatment is of great importance, as it helps to control classical and additional factors. Adequate eating habits, which may help reduce LDL-c levels in people with FH, are also important in treating and preventing additional risk factors such as systemic arterial hypertension, diabetes, obesity, oxidative stress, inflammatory process, and endothelial dysfunction, involved in the complex multifactorial mechanism of atherosclerosis.^4,12,13^

Among the dietary recommendations for FH, one of the few tested with a sample of individuals with this genetic disease is the possibility of reducing total cholesterol and LDL-c with phytosterol consumption, with most of the evidence coming from samples of children.^14,15^

The study by Radaelli et al.^8^ has great relevance and reinforces the need for constant searches for advances in treatment of FH individuals from childhood. Future studies should be conducted drug treatment and lifestyle changes jointly, considering dietary patterns and levels of physical activity, also little studied in children with FH. Adopting the best lifelong treatment may have benefits beyond lipid control, for example controlling comorbidities such as inflammation, obesity, and changes in blood pressure.
